# Correlation between HER-2/neu(erbB-2) expression level and therapeutic effect of combination treatment with HERCEPTIN and chemotherapeutic agents in gastric cancer cell lines

**DOI:** 10.1186/1475-2867-14-10

**Published:** 2014-01-29

**Authors:** Hai Cui, Ying Cheng, Su-Zhou Piao, Yun-Jie Xu, Hong-Hua Sun, Xian Cui, Xiang-Zi Li, Song-Nan Zhang, Long-Zhen Piao, Yong-Min Jin, Zhen-hua Lin, Xiong-Hu Shen

**Affiliations:** 1 Department of Oncology, Affiliated Hospital of Yanbian University, Yanji City, Jilin Province, People’s Republic of China; 2 Jilin Cancer Hospital, Changchun, China; 3 Department of Pathology, Yanbian University, Yanji, China

**Keywords:** HER-2/*neu*(erbB-2), HERCEPTIN, Gastric cancer, Combination treatment, CI

## Abstract

**Introduction:**

Although advanced gastric cancer has many limitations and response rate is marginal in chemotherapy. Overexpression of human epidermal growth factor receptor 2(HER-2/*neu*) gene and its protein are associated with increased cell division and a high rate of tumor growth and have been reported in several malignancies. Especially, approximately 30% of breast cancer patients have overexpression of HER-2/*neu* protein and the overexpression metastasize faster, induces resistance of the chemotherapy and down-regulate function of estrogen receptor. Recombinant humanized anti-HER2 antibody (Herceptin) inhibits proliferation of HER-2/*neu* overexpressing tumor cells and the use of that in combination in metastatic breast cancer have increased cytotoxicity of chemotherapeutic agents.

**Methods:**

We evaluated the expression of HER-2/*neu* protein in gastric cell lines by FACS and then comparing the cytotoxicity in chemotherapeutics (doxorubicin, cisplatin, paclitaxel, 5-FU) alone and in combination with Herceptin according to the expression of HER-2/*neu* protein by MTT assay.

**Results:**

1. NCI-N87 (88%) gastric cancer cell line and SK-BR-3 (89%) breast cancer cell line with strong positivity of HER-2/*neu* expression. YBC-2 (55%) and YBC-3 (48%) gastric cancer cell line with intermediated, weak positivity respectively. Negative control U-87 MG (6%) brain cancer cell line were showed low expression of HER-2/neu. 2. Cell growth was dose-dependently inhibited in HER-2/*neu* positive, control cell line SK-BR-3 by Herceptin treatment but not observed in HER-2/*neu* negative control cell line U-87 MG. Effective growth inhibition was not observed in gastric cancer cell lines with single treatment of Herceptin, all cell lines observed the dose-dependent growth inhibition to chemotherapeutic agents (doxorubicin, cisplatin, paclitaxel and 5-FU). 3. Combination of Herceptin with doxorubicin observed synergistic effects in all cancer cell lines except YBC-3, combination of Herceptin with cisplatin observed NCI-N87 and SK-BR-3 and combination of Herceptin with paclitaxel observed synergistic effects in YBC-2. Combination of Herceptin with 5-FU observed antagonistic effects in all cancer cell lines.

**Conclusions:**

According to HER-2/*neu* expression level, effect of anti-cancer agents was observed differently in combination of Herceptin with chemotherapeutic agents. This suggests that HER-2/*neu* expression level can be applied standard of combination drug selection in combination of Herceptin With chemotherapeutic agents in gastric cancer.

## Introduction

Since known that signal transduction pathways by growth factor and its receptor play an impotent role in the carcinogenic process many studies are being conducted. The HER-2/*neu*(erbB-2) protein, cell membrane glycoprotein, is a growth factor receptor has the activity of receptor tyrosine kinase [1]. HER2 has been frequently detected in a wide range of human tumors. Normal cell secretion associated with HER-2/neu protein, but destruction of normal cells control causing protein overexpression, increases the rate of cell division and growth induce precancerous changes [2].

The HER-2/*neu*(erbB-2) gene which is located on human chromosome 17q21 [3], which has partial homology with the epidermal growth factor receptor(EGFR) [4,5]. EGF (epidermal growth factor) receptor family has the activity of receptor tyrosine kinase and consisted of EGF receptor, c-erbB-2, c-erbB-3 and c-erbB-4 [6-8]. The HER-2/neu oncoprotein, encodes a p185kD membrane glycoprotein, receptor that is involved in the growth and diffentiation of the tumor cells and 25 ~ 30% overexpressed in breast, ovarian and gastric carcinomas [9-12] and the prognosis of these patients is poor [13-15].

Overexpression of HER-2/*neu* gene and it’s protein are associated breast cancer patient’s prognosis and therapy [16-18] and expression of HER-2/*neu* is strongly required for tumor growth [19]. Those suggest that HER-2/*neu* involved in the breast cancer occurrence, progression and malignant and HER-2/*neu* overexpression is more important than hormone receptors on the patients with lymph node metastasis [9]. Therefore, these therapeutic targets HER-2/*neu* protein drugs, recombinant humanized anti- HER-2/*neu* antibody - Herceptin, effective in the treatment of breast cancer has been reported [20].

Gastric cancer is the most prevalent cancer in the China. The occurrence of gastric cancer is the multistep carcinogenic process with variety genetic mutations. HER-2 status is correlated with the depth of invasion, TNM stage, lymph node and distant metastasis of gastric cancer [21]. HER-2/neu over-expression is related to poor prognosis of gastric cancer but has a modest effect on survival in gastric cancer as an independent prognosis factor [22]. Herceptin® as a cytostatic agent, the mono therapy efficient of antitumor effects cannot be expected but a lot of research is underway for combination therapy with other anticancer drugs or biologic agents. However, still not accurately study of combination therapy of Herceptin with anticancer drugs in gastric cell lines [13]. Therefore, in this study we investigate the effective combination of Herceptin (Trastuzumab, Genentech Inc, CA, USA) [23-25], targeting for HER-2*/neu* oncoprotein, with anti-cancer drugs in gastric cancer cell lines, depending on the HER-2*/neu* oncoprotein expression.

## Materials and methods

### Cells

Gastric cancer cell lines were used YBC-2, YBC-3 and NCI-N 87 cell lines. The YBC-2 and YBC-3 cell lines were established from intraperitoneal metastatic gastric cancer by Department of Pathology, Yanbian University. The NCI-N 87 cell line was established in the United State by NCI (National Cancer Institute, CRL5822, USA). The U-87 MG (ATCC, HTB14) brain tumor cell line and SK-BR-3 (ATCC, HTB30) breast cancer cell line were used for HER-2/*neu* protein expression positive and negative control, respectively.

These cell line were cultured use minimum essential media (MEM, GINCO BRL, Grand Island, NY, USA) which is containing inactivated (at 56°C for 30 min) 10% fetal bovine serum (FBS, GINCO BRL, Grand Island, NY, USA), 100 unit/ml Penicillin and 0.1 mg/ml Streptomycin. All culture incubations were performed in a humidified 37°C, 5% CO_2_ incubator.

### Drugs

Herceptin, Humanoid recombinant HER-2/neu antibody, was from USA (Trastuzumab, 440 mg/20 ml, Genentech Inc., San Francisco, CA, USA), and recognized anticancer drugs, effect in gastric cancer, Adriamycin (Doxorubicin, 10 mg/5 ml), Cisplatin (Cisplatin, 10 mg/vial), Taxol (Paclitaxel, 30 mg/vial) and 5-FU (5-Flourouracil, 250 mg/5 ml) were from China. When doing experiment each drug was used diluted in cell culture media.

### FACS analysis (HER-2/neu protein expression)

HER-2/*neu*(erbB-2) oncoprotein expression was done using Fluorescence-activated Cell Sorter (FACS, Becton Dickinson, U.S.A.) performed immunophenotyping in cell surface. Culture cells (1 × 10^6^) were harvested after treat with trypsin-EDTA (GIBCOBRL, Grand Island, NY, USA) and incubated with the primary antibody, Anti-HER-2/*neu* antibody (Mouse Monoclonal Antibody, LAB VISION, CA, USA), in a 1:200 dilution and 50 μl for 1 hour at 4°C. Stained cells were washed twice with cold PBS. Staining and washing were performed in PBS (phosphate-buffer solution, pH 7.6 Ca^++^ Mg^++^ free, GIBCO BRL, Grand Island, NY, USA) 2% FBS. After washing twice a secondary FITC-antimouse antibody (Anti-Mouse FITC-Conjugated antibody, Novocastra, USA) was used in a 1:40 dilution, and 50 μl were incubated for 40 minute at 4°C, after then washed twice with cold PBS. After completing the staining, filtration cell solution were used 40 um nylon mesh obtain the single cell suspended matter and doing analysis in the FACS.

### MTT assay (in vitro Herceptin and chemotherapeutic agents sensitivity test in gastric cancer cell lines)

The MTT assay has been used for *in vitro* drug sensitivity test in gastric cancer cell lines. MTT dye [3-(4,5-dimethyl thiazol-2-yl)-2,5-diphenyl tetrazolium bromide] is reduced to formazan (a dark purple water insoluble compound) by the succinate dehydrogenase system of active mitochondria, and hence, specifically used to the optical density (OD) of the wells was measured in an enzyme-linked immunosorbent assay (ELISA) reader Sunrise (TECAN) at 560 nm. The OD correlated linearly with the number of viable cells. Cells were plated at 180 ul media of 1 × 10^4^ cells per well in a 96 well multi-well plate and incubated for 24 h at 37°C and 5% CO_2_.

For single drug sensitivity test, two methods were applied. First, Herceptin (4.625, 9.25, 18.5, 37 μM), doxorubicin (0.0183, 0.183, 1.83, 18.3 μM), cisplatin (0.166, 1.666, 16.66, 166.6 μM), paclitaxel (0.0585, 0.585, 5.85, 58.5 μM) and 5-FU (38.43, 384.37, 3843.78, 38437.8 μM) were diluted in 20 μl media and added to the plates and incubated for 4 days. Control group were added only media without the drugs and incubated same durations. Second, the synergistic effect of the combination therapy with a combination of Herceptin + Doxorubicin, Herceptin + Cisplatin, Herceptin + Paclitaxel and Herceptin + 5- FU.

When combination treatment Herceptin and each anticancer drug performed sensitivity test, fixed molar concentration percentage of both drugs, after then adding 50 μl MTT, MTT was dissolved in phosphate buffer to the concentration of 2 mg/μl, added to each well and incubated 4 hours more. After removing the supernatant, placed 150 μl DMSO (Dimethyl sulfoxide, Sigma, USA) in each well and gently shaking about 15 minutes to dissolve the generated formazan. The optical density (OD) of the wells was measured in an enzyme-linked immunosorbent assay (ELISA) reader at 562 and 720 nm. The malignant cell survival was calculated by the equation (OD of drug well- OD of blank well/mean OD of control well - OD of blank well) × 100%. MTT assay was performed and repeated three times for each dose of the drug (5 well) the results were analyzed to obtain the average.

#### CI value calculation after combination therapy

CI (Combination index) was obtained by the formula of Chou-Talalay, was calculated from the value of IC50 (Dm) compared the efficacy and capacity curve (the m value). Classic formula of the Isobologram (CI = 1) was as follows.

$$\text{CI}={\left(D\right)}_{1}/{\left(\text{Dx}\right)}_{1}+{\left(D\right)}_{2}/{\left(\text{Dx}\right)}_{2}\;$$

Denominator of (Dx)_1_ and (Dx)_2_ were indicate the percentage cytotoxicity of when treated with single drug alone; numerator of (D)_1_ and (D)_2_ were indicate the percentage cytotoxicity of when treated with both drugs.

$$\text{Dx}=\text{Dm}{\left[\text{fa}/\left(1-\text{fa}\right)\right]}^{1m}$$

Dm is the median-effect dose (IC50), this calculated by median-effect plot of the X-intercept from the antilog.

$$X=\text{log}\left(D\right)\text{versus}\;Y=\text{log}\left[fa/\left(1-fa\right)\right]$$

m is the slope of the median-effect plot. Multiple drug effect analysis using Calcusyn (Biosoft, Cambridge, UK), were calculated values of m, Dm, Dx, and CI. (Dx) 1 and (Dx) 2 were calculated using the Chou-Talalay median-effect formula. CI <1, CI = 1, CI > 1 were called synergism, summation, and antagonism effects respectively.

### Statistical evaluation

Statistical analyses of the data were performed using the two-tailed Student *t*-test (SPSS 13.0 software; SPSS, Chicago, IL). *p* values less than 0.05 were considered statistically significant. Analysis of variance (ANOVA) was used for multiple group comparisons to determine the potential antitumor effects between groups.

## Results

### HER-2/neu expression in gastric cancer cell lines by FACS

**Figure 1:**
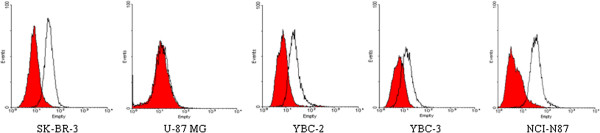
**HER-2/neu expression in gastric cancer cell lines.** The expression of HER2/neu protein in all cancer cell lines were measured by fluorescence-activated cell sorting analysis. The HER2/neu expression in three gastric cancer cell lines (YBC-2, YBC-3 and NCI-N87) were 48% (weak ), 55% (medium) and 88% (strong), respectively. All groups of Isotype control were not more than 0.5% (data not showed). As the positive control, SK-BR-3 of HER2/neu expression is 89%. U-87 MG showed lower expression of HER2/neu (6%) as negetive control.

The expression of HER2/neu protein was analysed in three different stomach cancer cell lines (YBC-2, YBC-3 and NCI-N87). The expression of this proteinis 48% (weak), 55% (medium) and 88% (strong) in YBC-2, YBC-3 and NCI-N87, respectively. The expression of this protein was 89% in positive breast cancer cell line SK-BR-3, and 6% in negative brain tumor cell line U-87 MG (Figure 1).

### The sensitivity of stomach cancer cell line to Herceptin and anti-cancer drug and the analysis of sensitivity to single drug treatment in vitro

**Figure 2:**
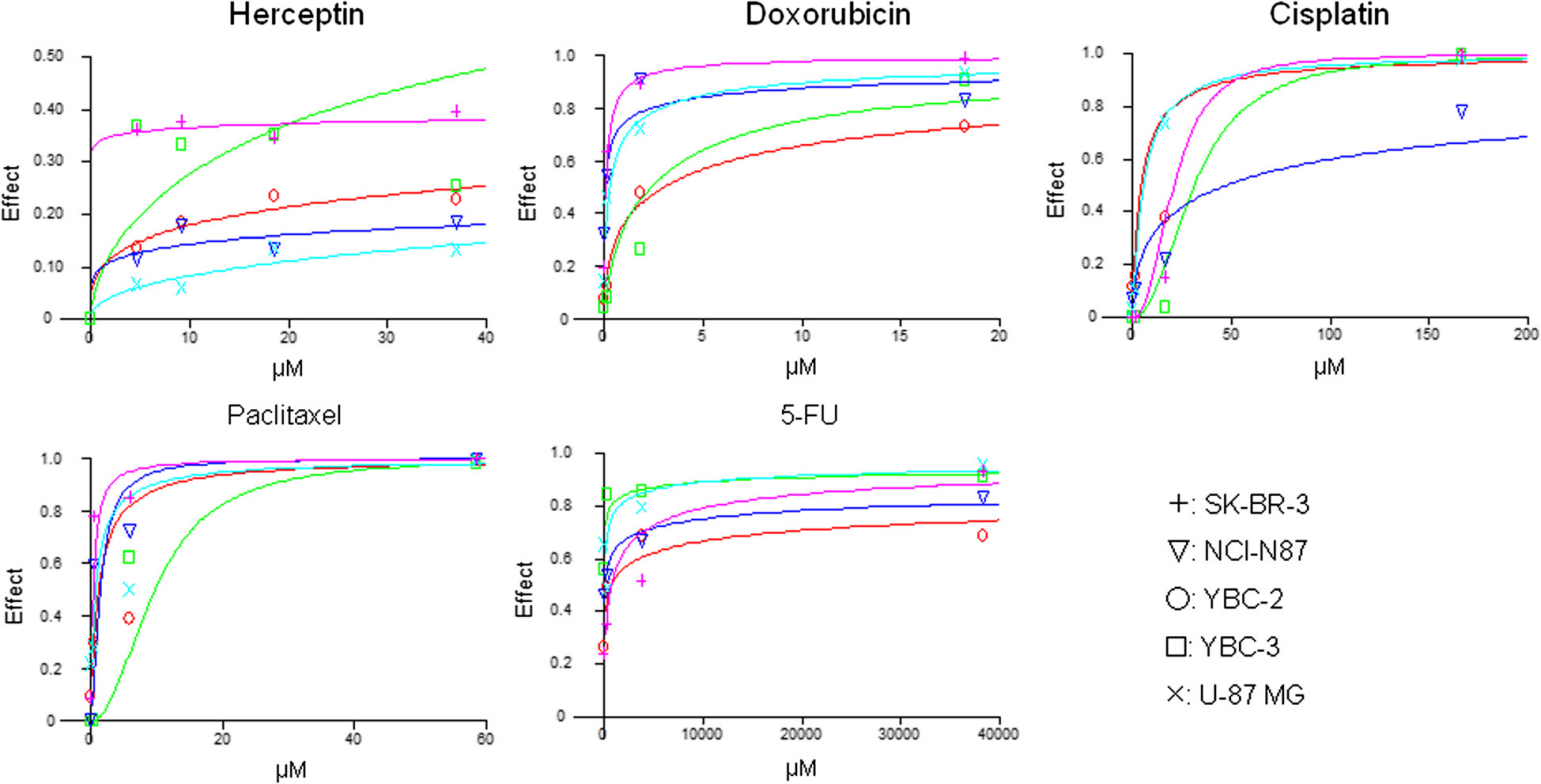
**Chemosensitivity of Herceptin® and four anti-cancer drugs in gastric cancer cell lines.** In single use of Herceptin, the arrest of cell growth was weak and IC_50_ was not detectable in vitro with stomach cancer cell line. In contrast, the four different anti-cancer drugs (doxorubincin, cisplatin, paclitaxel and 5-FU) showed dose-dependent inhibition of cell growth and IC_50_ was detected.

In single use of Herceptin, the arrest of cell growth was weak and IC_50_ was not detectable in vitro with stomach cancer cell line (Figure 2). In contrast, the four different anti-cancer drugs (doxorubincin, cisplatin, paclitaxel and 5-FU) showed dose-dependent inhibition of cell growth and IC_50_ was detected (Table 1, Figure 2).

**Table 1:** IC30 and IC50 of five anti-cancer agents against YBC-2, YBC-3, NCI-N87, U-87 MG and SK-BR-3 cell lines

**Anticancer agents**	**Cell lines (IC**_**30**_**) *μM***				
	**YBC-2**	**YBC-3**	**NCI-N87**	**SK-BR-3**	**U-87 MG**
Herceptin	82.36	11.83	1326.72	0.02	289.09
Doxorubicin	0.6	0.76	0.01	0.03	0.07
Cisplatin	1.79	21	9.28	14.54	2.58
Paclitaxel	0.52	6.45	0.86	0.19	0.3
5-FU	33.11	0.22	4.11	148.75	5.51
**Anticancer agents**	**Cell lines (IC**_**50**_**) *μM***				
	**YBC-2**	**YBC-3**	**NCI-N87**	**SK-BR-3**	**U-87 MG**
Doxorubicin	2.93	2.31	0.07	0.1	0.29
Cisplatin	4.51	30.73	45.86	5.65	20.63
Paclitaxel	1.24	9.61	1.49	0.42	0.79
5-FU	752.04	4.74	119.61	753.42	46.58

### The effect of the combination of Herceptin and anti-cancer drugs

**Figure 3:**
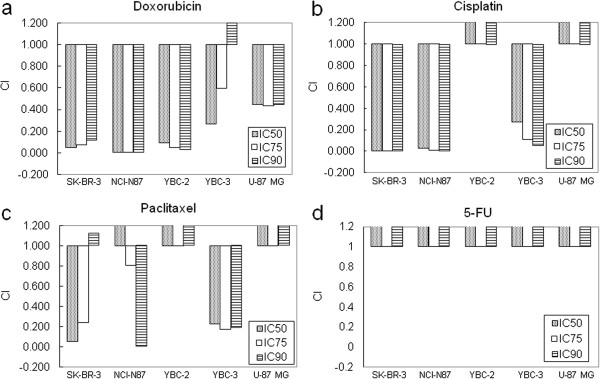
**Combination Index as a growth inhibitory effect of combination treatment with Herceptin® and anti-cancer agents. a**. In the combination of Herceptin and doxorubincin treatment, the synergetic effect was observed in YBC-2 and NCI-N87 cells, but not in YBC-3 cell line. **b**. In the combination of herceptin and cisplatin, the synergetic effects were observed in SK-BR-3, NCI-N87 and YBC-3 cell lines (CI < 1). However, the inhibitory effects were observed in YBC-2 and U-87 MG cell lines (CI > 1). **c**. The treatment of Herceptin and paclitaxel, in YBC-3 and U-87 MG cell lines, the inhibitory effects were observed (CI > 1), however, in YBC-3 cell line, the synergetic effect was observed (CI < 1). In SK-BR-3 and NCI-87 cell lines, the effects were different depends on the dose of the drugs. **d**. In the combination of Herceptin and 5-FU, the inhibitory effects were observed in all of the cell lines (CI > 1).

In the combination of Herceptin and doxorubincin treatment, the synergetic effect was observed in YBC-2 and NCI-N87 cells, but not in YBC-3 cell line which has weak expression of HER2*/neu* protein (Table 2, Figure 3a, *P* < 0.05). In the combination of herceptin and cisplatin, the synergetic effects were observed in SK-BR-3, NCI-N87 and YBC-3 cell lines (CI < 1, *P* < 0.05). However, the inhibitory effects were observed in YBC-2 and U-87 MG cell lines (Combination Index > 1, *P* < 0.05) (Table 3, Figure 3b). The treatment of Herceptin and paclitaxel, in YBC-3 and U-87 MG cell lines, the inhibitory effects were observed (CI > 1), however, in YBC-3 cell line, the synergetic effect was observed (CI < 1, *P* < 0.05). In SK-BR-3 and NCI-87 cell lines, the effects were different depends on the dose of the drugs (Table 4, Figure 3c). In the combination of Herceptin and 5-FU, the inhibitory effects were observed in all of the cell lines (CI > 1, *P* < 0.05) (Table 5, Figure 3d).

**Table 2:** Calculated values for the Combination Index as a growth inhibitory effect of combination treatment with Herceptin® and Doxorubicin in gastric cancer cell lines

**YBC-2**	**Combination Index Value**			**Parameter (μM)**		
**Drug**	**ED50**	**ED75**	**ED90**	**Dm**	**m**	**r**
**DOX**				2.93	0.54	0.98
**HER**				1201.02	0.32	0.91
**DOX + HER**	0.09	0.05	0.03	0.25	0.74	0.96
**Diagnosis combined effect**	synergy	synergy	synergy			
**YBC-3**	**Combination Index Value**			**Parameter (μM)**		
**Drug**	**ED50**	**ED75**	**ED90**	**Dm**	**m**	**r**
**DOX**				2.310	0.768	0.946
**HER**				46.063	0.623	0.970
**DOX + HER**	0.266	0.598	1.377	0.406	0.469	0.925
**Diagnosis combined effect**	synergy	synergy	antagonism			
**NCI-N87**	**Combination Index Value**			**Parameter (μM)**		
**Drug**	**ED50**	**ED75**	**ED90**	**Dm**	**m**	**r**
**DOX**				0.070	0.398	0.864
**HER**				113350	0.191	0.635
**DOX + HER**	0.005	0.004	0.003	0.000	0.437	0.995
**Diagnosis combined effect**	synergy	synergy	synergy			
**U-87 MG**	**Combination Index Value**			**Parameter (μM)**		
**Drug**	**ED50**	**ED75**	**ED90**	**Dm**	**m**	**r**
**DOX**				0.295	0.620	0.997
**HER**				1845.5	0.457	0.860
**DOX + HER**	0.441	0.435	0.448	0.101	0.589	0.992
**Diagnosis combined effect**	synergy	synergy	synergy			
**SK-BR-3**	**Combination Index Value**			**Parameter (μM)**		
**Drug**	**ED50**	**ED75**	**ED90**	**Dm**	**m**	**r**
**DOX**				0.103	0.832	0.998
**HER**				1424000	0.046	0.435
**DOX + HER**	0.046	0.074	0.117	0.005	0.616	0.985
**Diagnosis combined effect**	synergy	synergy	synergy			

*DOX*, Doxorubicin; *HER*, Herceptin.

**Table 3:** Calculated values for the Combination Index as a growth inhibitory effect of combination treatment with Herceptin® and Cisplatin in gastric cancer cell lines

**YBC-2**	**Combination Index Value**			**Parameter (μM)**		
**Drug**	**ED50**	**ED75**	**ED90**	**Dm**	**m**	**r**
**CDDP**				4.512	0.921	0.882
**HER**				1201.0	0.316	0.915
**CDDP + HER**	1.375	2.707	5.337	2.232	0.587	0.965
**Diagnosis combined effect**	antagonism	antagonism	antagonism			
**YBC-3**	**Combination Index Value**			**Parameter (μM)**		
**Drug**	**ED50**	**ED75**	**ED90**	**Dm**	**m**	**r**
**CDDP**				30.733	2.227	0.929
**HER**				46.063	0.623	0.970
**CDDP + HER**	0.274	0.110	0.054	12.091	1.472	0.998
**Diagnosis combined effect**	synergy	synergy	synergy			
**NCI-N87**	**Combination Index Value**			**Parameter (μM)**		
**Drug**	**ED50**	**ED75**	**ED90**	**Dm**	**m**	**r**
**CDDP**				45.86	0.53	0.93
**HER**				1.13E + 05	0.19	0.63
**CDDP + HER**	0.03	0.01	0.01	0.83	0.88	0.96
**Diagnosis combined effect**	synergy	synergy	synergy			
**U-87 MG**	**Combination Index Value**			**Parameter (μM)**		
**Drug**	**ED50**	**ED75**	**ED90**	**Dm**	**m**	**r**
**CDDP**				5.65	1.08	0.98
**HER**				1845.53	0.46	0.86
**CDDP + HER**	9.18	9.85	10.58	2.56	1.01	0.99
**Diagnosis combined effect**	antagonism	antagonism	antagonism			
**SK-BR-3**	**Combination Index Value**			**Parameter (μM)**		
**Drug**	**ED50**	**ED75**	**ED90**	**Dm**	**m**	**r**
**CDDP**				20.63	2.42	0.94
**HER**				1.42E + 06	0.05	0.43
**CDDP + HER**	0	0	0	1.45	1.44	0.99
**Diagnosis combined effect**	synergy	synergy	synergy			

*CDDP*, Cisplatin; *HER*, Herceptin.

**Table 4:** Calculated values for the Combination Index as a growth inhibitory effect of combination treatment with Herceptin® and Paclitaxel in gastric cancer cell lines

**YBC-2**	**Combination Index Value**			**Parameter (μM)**		
**Drug**	**ED50**	**ED75**	**ED90**	**Dm**	**m**	**r**
**TAX**				1.24	0.98	0.9
**HER**				1201.02	0.32	0.92
**TAX + HER**	6.03	14.77	36.21	0.85	0.54	0.82
**Diagnosis combined effect**	antagonism	antagonism	antagonism			
**YBC-3**	**Combination Index Value**			**Parameter (μM)**		
**Drug**	**ED50**	**ED75**	**ED90**	**Dm**	**m**	**r**
**TAX**				9.609	2.129	0.940
**HER**				46.063	0.623	0.970
**TAX + HER**	0.225	0.172	0.185	7.010	1.220	0.889
**Diagnosis combined effect**	synergy	synergy	synergy			
**NCI-N87**	**Combination Index Value**			**Parameter (μM)**		
**Drug**	**ED50**	**ED75**	**ED90**	**Dm**	**m**	**r**
**TAX**				1.49	1.56	0.96
**HER**				113350	0.19	0.63
**TAX + HER**	1747.94	0.81	0	858.42	−0.16	0.90
**Diagnosis combined effect**	antagonism	synergy	synergy			
**U-87 MG**	**Combination Index Value**			**Parameter (μM)**		
**Drug**	**ED50**	**ED75**	**ED90**	**Dm**	**m**	**r**
**TAX**				0.79	0.89	0.87
**HER**				1845.53	0.46	0.86
**TAX + HER**	361.02	1571.47	6840.35	1.27	0.41	0.89
**Diagnosis combined effect**	antagonism	antagonism	antagonism			
**SK-BR-3**	**Combination Index Value**			**Parameter (μM)**		
**Drug**	**ED50**	**ED75**	**ED90**	**Dm**	**m**	**r**
**TAX**				0.42	1.11	0.96
**HER**				1.42E + 06	0.05	0.43
**TAX + HER**	0.05	0.24	1.12	0	0.44	0.88
**Diagnosis combined effect**	synergy	synergy	antagonism			

*TAX*, Paclitaxel; *HER*, Herceptin.

**Table 5:** Calculated values for the Combination Index as a growth inhibitory effect of combination treatment with Herceptin® and 5-FU in gastric cancer cell lines

**YBC-2**	**Combination Index value**			**Parameter (μM)**		
**Drug**	**ED50**	**ED75**	**ED90**	**Dm**	**m**	**r**
**5-FU**				752.04	0.27	0.94
**HER**				1201.02	0.32	0.91
**5-FU + HER**	8060.81	1620.97	325.97	285.29	0.45	1
**Diagnosis combined effect**	Antagonism	Antagonism	Antagonism			
**YBC-3**	**Combination Index value**			**Parameter (μM)**		
**Drug**	**ED50**	**ED75**	**ED90**	**Dm**	**m**	**r**
**5-FU**				4.739	0.279	0.920
**HER**				46.063	0.623	0.970
**5-FU + HER**	75054	2.89E + 05	1.13E +06	71.077	0.207	0.928
**Diagnosis combined effect**	antagonism	antagonism	antagonism			
**NCI-N87 **	**Combination Index value**			**Parameter (μM)**		
**Drug**	**ED50**	**ED75**	**ED90**	**Dm**	**m**	**r**
**5-FU**				119.61	0.25	0.97
**HER**				1.1E + 05	0.19	0.63
**5-FU + HER**	1.9E + 17	3.2E + 02	5.3E +29	3.7E + 14	−0.02	0.87
**Diagnosis combined effect**	antagonism	antagonism	antagonism			
**U-87 MG **	**Combination Index value**			**Parameter (μM)**		
**Drug**	**ED50**	**ED75**	**ED90**	**Dm**	**m**	**r**
**5-FU**				46.58	0.4	0.84
**HER**				1845.53	0.46	0.86
**5-FU + HER**	25540	4940.8	955.81	6.48	0.98	1
**Diagnosis combined effect**	antagonism	antagonism	antagonism			
**SK-BR-3**	**Combination Index value**			**Parameter (μM)**		
**Drug**	**ED50**	**ED75**	**ED90**	**Dm**	**m**	**r**
**5-FU**				753.42	0.52	0.93
**HER**				1.42E + 06	0.05	0.43
**5-FU + HER**	226.52	173.36	132.68	488.15	0.6	0.93
**Diagnosis combined effect**	antagonism	antagonism	antagonism			

*DOX*, Doxorubicin; *HER*, Herceptin; *CI* Combination index.

## Discussion

The overexpression of the growth factors or the receptors of the growth factors in the signaling pathways, will not only highly affect cell growth but also induce tumorigenesis. Pro-oncogenes play important roles during cell growth, development and differentiation. They also involved in the function of the growth factors and their receptors, or other signaling pathways and induce the abnormal cell growth and induce the cells become tumor cells. The overexpression of receptors of growth factors, such as FGF-5, TGF-a, EGF, will induce tumorigenesis.

After HER-2/neu protein was first discovered in breast cancer cells [4], this protein has been actively studied in breast cancer field. In about 30% of total breast cancer patients, Her-2/neu is overexpressed. Many in vitro and in vivo studies have shown that the increased level of HER2 was associated with the early stage breast cancer [26], recurrence rates, cancer metastasis and the decreased efficiency of hormone-based therapy [5,27]. HER-2 has been considered as factor for poor prognosis. HER-2 oncogene is located on the chromosome seventeen q21 and structurally has close relationship with EGFR gene which is locate in chrosome seven and v-erbB oncogene. Point mutation of HER-2 in transmembrane domain forms 4.8 kb mRNA and 185 Kd glycoprotein which belongs to tyrosine kinases.

Overexpression of HER-2 protein is not discovered in normal tissues. In contrast, it is overexpressed in primary and metastatic cancer tissues which have been considered as the target of therapy. HER-2/neu might be a useful target for immunotherapy in colorectal carcinoma, anti-HER2 anti-CD3 BsAb exerting clear anti-tumor effects [28]. In vitro and in vivo studies showed that inhibition of HER-2 with anti-HER2 antibody showed high toxicity to breast cancer cells [29]. Herceptin is anti-HER2 antibody. It was developed as the combination of human immunoglobulin with murine antigen and can be used in the human. The results from clinical trial with metastic breast cancer patients showed that 21% efficacy and also increased the efficiency of anticancer drugs [30]. Park et al. reported that HER-2 gene has close relationship with stomach cancer formation and Yonemura et al. suggested that overexpression of HER2 is related to poor prognosis in stomach cancer patients and anti-HER2 antibody may be an essential drug for the therapy [31].

The most efficient therapy of the stomach cancer is surgery. However, recurrence or metastatic recurrence has been found in 80% patients after surgery, alternative anti-cancer therapy or induced anti-cancer therapy is necessary for the patients after surgery. However, the chemical drugs which have been used in these patients, such as 5-FU, doxorubincin, cisplatin, mitomycin and paclitaxel, showed only 15-30% efficiency, it is essential to understand the mechanism of stomach cancer formation and develop a new therapy.

In this study, first we analyzed the expression of Her-2 in three stomach cancer cell lines (YBC-2, YBC-3, NCI-N87) and found that HER2 is highly expressed in NCI-N87. In YBC-3, the expression of HER2 is much weaker and in YBC-2, the expression of this protein is higher than that in YBC-2 but lower than that in NCI-N87. To more clearly analyze the expression of HER-2 in the cells, flow cytometry was used. Doxorubin, cisplatin, paclitaxel and 5-FU four different anticancer drugs were commonly used in chemotherapy and their effects are relatively effective and these compounds have synergic effects when combined with Herceptin in breast cancer therapy.

In SK-BR-3, the growth inhibitory effect of Herceptin IC40 was observed. This can be explained that the half-life of Herceptin is short (72 hrs), the compound was degraded and not present anymore during MTT assay (96 hrs). Also Herceptin is a cytostatic agent which binds to the growth factor receptor. Although it inhibits HER-2 mediated cell growth pathway, it may induce cell growth through other pathways which there was no difference after increase the dose of compound. In our work, the maximum dose of Herceptin was used and this dose was known effective in breast cancer treatment.

It was found that all of the four compounds could inhibit the growth of cells dose-dependently in SK-BR-3 cells where HER-2 is highly expressed. This data confirmed the relationship between HER-2 expression and doxorubicin efficiency. However, cisplatin didn’t dose-dependently inhibit cell growth in HER-2 highly expressed NCI-N87 stomach cancer cells. In contrast, it inhibited cell growth in other cell lines. Different from cisplatin and Herceptin, paclitaxel inhibited the growth of cells independent of HER-2 expression. Yu et al. [32]. showed that the cells developed resistant to paclitaxel and Baselga et al. [33]. Showed drugs inhibition need more detailed research is needed.

The synergism, summation and antagonism among Herceptin and other anti-cancer compound is not depends on the expression of HER-2 or the change of phosphorylation [20]. Herceptin single drug has no cytotoxicity in cancer cell in vitro. But by blocking its downstream signaling pathways, to inhibit the cell survival or drug resistance gene expression to achieve synergistic effect in combination therapy. According to previous reports, pretreatment of doxorubicin can increase the expression of EGFR or TGF-α in cells, therefore the combined with anti -EGFR antibody treatment maybe achieve synergy effect in most of cell lines including U87MG cells even that has no or little HER2 expression. This needs forther studies to confirm. Cisplatin induces DNA adduct and inhibits cell growth. It disrupts DNA replication during cell division. If pretreatment the cells with anti-HER2 antibody, the repair efficiency of the DNA breaks which induced by cisplatin is decreased [25]. Paclitaxel also showed dose-dependent efficiency. Chang et al. [31]. showed that paclitaxel causes G2/M phase arrest and also induces cell toxicity in G0/G1 phase [34]. When combination with herceptin, the effect of paclitaxel on G2/M phase arrest was more efficient and increased the toxicity to the cells. Also Herceptin treatment increased G0/G1 phase cells [35] which increased the sensitivity of the cells to paclitaxel treatment. In the treatment of Herceptin and 5-FU, all the cells showed resistant effect (CI > 1).

In conclusion, in different type of stomach cancer cell lines, the expression of HER-2 protein is different, so different anti-cancer compounds (doxorubicin, cisplatin, paclitaxel and 5-FU) should be used depends on the expression of HER-2. This suggests that HER-2/*neu* expression level can be applied to standard for drug selection in combination of Herceptin® with chemotherapeutic agents in gastric cancer.

## Competing interests

The authors declare that they have no competing interests.

## Authors’ contributions

HC performed part of the experiments, participate d in the coordination of the study, and wrote the final manuscript. AHJ performed the main part of the experiments. SZP participated in the experiments and in the coordination of the study. HHS, XC,SNZ, LZP, and YMJ analyzed the data and participated in the experiments. ZHL reviewed the entire manuscript. ZHL and XHS conceived and designed the study, analyzed the data and helped to draft the manuscript. XHS was responsible for histological examination. All authors read and approved the manuscript.
